# Sustained organic amendments utilization enhances ratoon crop growth and soil quality by enriching beneficial metabolites and suppressing pathogenic bacteria

**DOI:** 10.3389/fpls.2023.1273546

**Published:** 2023-09-18

**Authors:** Nyumah Fallah, Ziqin Pang, Zhaoli Lin, Witness Joseph Nyimbo, Wenxiong Lin, Sylvain Ntambo Mbuya, Captoline Ishimwe, Hua Zhang

**Affiliations:** ^1^ Key Laboratory of Sugarcane Biology and Genetic Breeding, Ministry of Agriculture, Fujian Agriculture and Forestry University, Fuzhou, China; ^2^ Fujian Provincial Key Laboratory of Agro-ecological Processing and Safety Monitoring, College of Life Sciences, Fujian Agriculture and Forestry University, Fuzhou, China; ^3^ Laboratoire de Recherche en Biofortification, Defense et Valorisation des Cultures (BioDev), Département de Production Végétale, Faculté des Sciences Agronomiques, Université de Lubumbashi, Lubumbashi, Democratic Republic of Congo; ^4^ Key Laboratory of Ministry of Education for Genetics, Breeding and Multiple Utilization of Crops/Fujian Key Laboratory for Crop Breeding by Design, Fujian Agriculture and Forestry University, Fuzhou, China; ^5^ Experiment Station of Ministry of Agriculture and Rural Affairs for Jute and Kenaf in Southeast China/Fujian Public Platform for Germplasm Resources of Bast Fiber Crops, Fujian Agriculture and Forestry University, Fuzhou, China

**Keywords:** metabolites, filter mud, biochar, disease-causing bacteria, soil quality, ratoon crop

## Abstract

**Introduction:**

Organic soil amendments such as filter mud (FM) and biochar (BC) can potentially influence the abundance and composition of metabolites. However, our current understanding of the stimulatory effects of FM and BC’s long-term impact on stress-regulating metabolites, such as abscisic acid (ABA), jasmonic acid (JA), melatonin, and phenyllactic acid (PLA), and these substrates regulatory effects on disease-causing bacteria in sugarcane ratooning field, which is susceptible to nutrients depletion, diseases, etc., remain poorly understood. Additionally, little is known about how the long-term interaction of these substrates and compounds influences sugarcane ratooning soil enzyme activities, nutrient cycling, and crop growth performance.

**Methods:**

To answer these questions, we adopted metabolomics tools combined with high-throughput sequencing to explore the stimulatory effects of the long-term addition of FM and BC on metabolites (e.g., PLA and abscisic aldehyde) and quantify these substrates’ regulatory effects on disease-causing bacteria, soil enzyme activities, nutrient cycling, and crop growth performance.

**Results:**

The result revealed that ratoon crop weight, stem diameter, sugar content, as well as soil physico-chemical properties, including soil nitrate (NH_3_^+^-N), organic matter (OM), total nitrogen (TN), total carbon (TC), and β-glucosidase, marked a significant increase under the BC and FM-amended soils. Whereas soil available potassium (AK), NO_3_^–^N, cellulase activity, and phosphatase peaked under the BC-amended soil, primarily due to the enduring effects of these substrates and metabolites. Furthermore, BC and FM-amended soils enriched specific stress-regulating metabolites, including JA, melatonin, abscisic aldehyde, etc. The sustained effects of both BC and FM-amended soils suppressed disease-causing bacteria, eventually promoting ratooning soil growth conditions. A number of key bioactive compounds had distinct associations with several beneficial bacteria and soil physico-chemical properties.

**Discussion:**

This study proves that long-term BC and FM application is one of the eco-friendly strategies to promote ratoon crop growth and soil quality through the enrichment of stress-regulating metabolites and the suppression of disease-causing bacteria.

## 1 Introduction

Ratooning is a farming practice that involves harvesting a monocot crop by cutting most of the aboveground portions but leaving the roots and the growing shoot apices undamaged to allow the crops to recover and produce new crops in the subsequent season (Bamisile et al., 2021; Fallah et al., 2023). This farming practice is common among several crops, including sugarcane, rice, banana, etc. It has been pointed out that sugarcane ratoon farming practice represents about 50% of the overall sugarcane production globally (Li and Yang, 2015). However, this farming practice is confronted with a number of soil constraints or environmental stressors (soil-borne diseases, high pH, etc.) primarily due to the indiscriminate long-term use of pesticides, insecticides, synthetic fertilizers, etc., to maintain optimum crop growth and yield (Diacono and Montemurro, 2011). Considering these concerns, it is of the essence to adopt ameliorative soil management strategies that can mitigate this problem. It is relevant to mention that organic amendments such as filter mud (FM) and biochar (BC) offer a huge potential in this regard.

It has been reported that BC is a carbonaceous solid residue generated by thermochemical treatments, including pyrolysis of biomass materials and gasification under oxygen-limited conditions. Its distinct characteristics (e.g., pore structure, low solubility, abundant surface-active groups, high specific surface area, and strong electrical conductivity) offer potential benefits in promoting soil health, soil fertility, crop production, carbon sequestration, etc. Zhang et al. (2017) revealed that BC application enhanced soil fertility under simulated nitrogen deposition. Knoblauch et al. (2021) also mentioned that BC utilization positively influenced crop yields and plant available nutrients. Mounting evidence has revealed that novel substrates can alter soil microbial abundance and community (Pang et al., 2022). Zhu et al. (2022) reported that the addition of BC was crucial in increasing soil microbial biomass, crop yield, and soil fertility.

Correspondingly, an increasing number of reports have shed light on the importance of FM application in agriculture as a vital source of major plant nutrients, including nitrogen, phosphorus, and potassium (Barry et al., 2001; Shaaban et al., 2022). Few studies have also documented the impacts of FM on soil microbial community and abundance (Abubakar et al., 2022). We recently reported that FM utilization significantly promoted *Anabaena* and *Enterobacter*. A related study documented that the application FM triggered a linear increase in microbial biomass (Rasul et al., 2008). Given the crucial roles these substrates play in shaping vital soil attributes (e.g., soil nutrients and the microbial community), it is essential to expand our understanding of how prolonged use of both BC and FM influences other soil parameters (e.g., metabolomics).

The growth and development of plants are characterized by the excretion of an array of bioactive compounds in soils (Ma et al., 2022), also referred to as metabolites. These metabolites can serve as chemical repellants and attractants in the rhizosphere zone (More, 2020). Metabolites are deemed major transporters for material cycling and energy exchange. Furthermore, they are also regarded as information carriers between soil ecosystem and the belowground compartment of plants (Huang et al., 2020). Additionally, the excretion of metabolites in soils can impact soil physico-chemical characteristics, especially in the rhizosphere zone (Wen et al., 2022). Panchal et al. (2022) recently pointed out that plant metabolites were crucial in retaining the integrity of soil biogeochemical cycles, namely, the carbon cycle. A related study documented that metabolites played a crucial role in altering the transport properties of plant rhizosphere soil (Paporisch et al., 2021). Furthermore, a growing body of research has reported that the rhizosphere zone is one of the dynamic interfaces in terrestrial environments, making it an ideal habitat for the interactions of plant-soil-microbe and biological activities (Hinsinger et al., 2005), which is essential for material cycling in terrestrial ecosystems. Song et al. (2020) and Hu et al. (2018) studies showed that plant metabolites exhibited structural effects on microbial communities, diversity, and functions. Despite these findings, our current understanding of the stimulatory effects of the long-term addition of both FM and BC on metabolites, especially those deemed as stress regulators, and these substrates’ regulatory effects on disease-causing bacteria in sugarcane ratooning field, which is susceptible to nutrients depletion, insect pests, diseases, etc., remain poorly understood. Additionally, little is known about how these compounds and substrates long-term interaction influences ratooning soil enzyme activities, nutrient cycling, and crop growth performance. To answer these questions, we adopted metabolomics tools combined with high-throughput sequencing to explore the stimulatory effects of long-term addition of FM and BC on metabolites (e.g., PLA and abscisic aldehyde), and quantify these substrates regulatory effects on disease-causing bacteria, soil enzyme activities, nutrient cycling, and crop growth performance.

## 2 Materials and methods

Samplings were conducted in January 2021 from a long-term ratoon sugarcane field trial set up in March 2019 at Fujian Agriculture and Forestry University in Fuzhou, Fujian Province (latitude 26°5′0′′ east longitude 119°13′47′′). The climatic condition of the site features a humid subtropical climate with rainfall of 1369 mm and an annual average temperature of 20°C. Additional information about the sites is provided in **Table 1** below. The field trial was set up in a randomized block design consisting of three treatments: biochar applied at the rate of 20 t ha^-1^ (BC), filter mud applied at the rate of 20 t ha^-1^ (FM), and control (CK). We replicated each treatment three times, with each covering a plot of 36 m^2^ (6 m x 6 m). The soil was plowed at a depth of 30 cm using a rotary tiller. The BC and FM-amended soils were applied evenly and mixed before cultivating the sugarcane. After that, the BC and FM fields were supplemented with 375 kg of compound fertilizer (N-P_2_O_5_-K_2_O 15-15-15), while the CK field was supplemented with 375 kg/hm^2^ of compound fertilizer (N-P_2_O_5_-K_2_O 15-15-15).

**Table 1:** Initial properties of the soils, biochar, and filter mud.

Parameters	Soil properties	Biochar properties	Filter mud properties
TC g (kg-^1^)	7.22	35.08	11.21
TN (kg-^1^)	3.54	1.27	2.01
pH	8.89	11.01	10.22
AK (kg-^1^)	9.01	17.03	4.99
AP (kg-^1^)	13.11	5.21	7.11
OM (kg-^1^)	16.22	9.44	14.21
EC (dS m-^1^)	3.11	3.21	0.99
C/N	2.04	7.15	5.58

### 2.1 Root tissue and rhizosphere soil sampling and preparation

Three sugarcane plants were randomly selected from each plot. These plants were gently extracted from the soil and shaken to remove excess soil, dirt, and other impurities attached to the plant root system. After that, the roots were rinsed using sterile water. The cleaned roots were cut off (∼100 g) with a sterile scalpel into 50 mL sterile centrifuge tubes and stored in liquid nitrogen. The rhizosphere soils were sampled around the sugarcane roots with a standard soil ring knife. Finally, 18 samples were generated. The samples were placed into 50 mL sterile centrifuge tubes, stored in liquid nitrogen, transferred to the laboratory, and stored at -80°C.

### 2.2 Assessment of ratoon crop traits and soil physico-chemical characteristics

The crop weight was determined by calculating the fresh weight of 25 sugarcanes. The crop height was evaluated using a meter rod from the soil surface to the top of the crop. The crop height was assessed using a meter rod extending from the soil surface to the top of the crop. Moreover, we used a Vernier caliper to determine the stalk diameter around the middle of the crop stalk. The sugar content of the crop was assessed using Polartronic M 202 TOUCH (589 + 882 nm: SCHMIDT+HAENSCH GmbH & Co., Berlin, Germany) equipment.

Soil pH was tested with Sartorius PB-10 (Germany) (1:2.5 soil/water suspensions) (Bi et al., 2010). Walkley-black approach was used to estimate soil organic matter content. Soil AK was measured using an H_2_SO_4_-H_2_O_2_ flame photometer. Soil TC and TN were investigated using the approach documented by Bao (2000). Molybdenum Blue method was used to evaluate soil AP. Soil NH_3_^+^-N and NH_4_^+^-N were assessed with 2.0 M KCl and measured using the continuous flow analyzer (San++, Skalar, Holland). We adopted the methods documented in the study conducted by Sun et al. (2014) to investigate soil enzyme activities. Briefly, the investigation of urease activity was conducted based on the NH_4_^+^-N released during the incubation of the samples with 10 mL of 10% urea solution and 20 mL of citric acid buffer (pH 6.7) at 37°C (24 h). We evaluated phosphatase activity by incubating the samples with 0.25 mL of toluene and 1 mL of disodium p-nitrophenyl phosphate tetrahydrate and placed in a water bath at 37°C (1 h). In addition, β-glucosidase enzyme activity was determined by incubating the samples with 50 mM cellobiose substrate solution in citrate-phosphate buffer (pH 6.30) in a shaker at 37°C (1 h). Cellulase activity involved the determination of reducing sugars produced when medium samples were incubated with acetate buffer (50 mM, pH 5.5), carboxymethyl cellulose, and toluene at 37°C (24 h).

### 2.3 DNA extraction, PCR amplification, 16S rRNA sequencing, and data processing

Fresh soil (0.5 g) was used to extract genomic DNA using Fast DNA™ Spin Kit (MP Biomedicals, LLC, Santa Ana, USA). DNA quantity and quality assessment were performed by computing their absorbance (A260 and 280 nm) using BioTek Synergy H1 Hybrid Multi-Mode Microplate Reader (BioTek, USA). We used 50-µL, 1 mM dNTPs (deoxynucleoside triphosphate), 1 U of Platinum Taq, 1 × PCR buffer, and a primer at 5 µM and DNA template (10 ng) to conduct PCR analysis. PCR amplification was carried out with an initial denaturation at 94°C (3 min), 5 cycles of denaturation at 94°C (30 s), annealing at 45°C (20 s), and extension at 65°C (30 s). The process was later followed by 20 cycles of denaturation at 94°C (20 s), annealing at 55°C (20 s), extension at 72°C (30 s), and an extension at 72°C (5 min). Lastly, we leveraged Illumina Hiseq 2500 platform (2 × 250 paired ends) of Biomarker Technologies Corporation, Beijing, China, to perform 16S rRNA sequencing. We deposited the raw data on the NCBI Sequence Read Archive platform (accession no. PRJNA929962).

Following the barcode apportioned to every sample, we merged the original paired-end reads of DNA fragments with FLASH (Tan et al., 2017). Sequence clustering was carried out using operational taxonomic units (OUTs) at 97% similarity. Sequences were selected to annotate the taxonomic data by employing the Ribosomal Database Project (RDP). Primers with nucleotides ≥ 200 or low-quality sequences and a high average quality score (Q ≥ 20) were removed and clustered at 97% nucleotide similarity. Taxonomic classification was performed with SILVA database (SILVA Release 138, Bacteria). Lastly, bioinformatics analysis was conducted using biomarker biocloud platform (www.biocloud.net).

### 2.4 Metabolites extraction, LC-MS/MS analyses, processing, and annotation

A mixer mill (MM400, Retsch) was used to ground the samples into fine powders (1.5 min) at 30 Hz. Lyophilized powder (100 mg) was used to extract metabolites at 4°C using 0.8 ml of 70% aqueous methanol (methanol: H_2_O_2_, 70:30, v/v). After adding pure methanol, centrifugation was conducted for 10 min (10000 g). We gathered, homogenized, and sieved the supernatants accordingly (SCAA-104, 0.22 mm pore size; ANPEL Shanghai, China, www.anpel.com.cn/). Samples were mixed into tissue-specific samples, namely root tissue and rhizosphere soil, to assess the inter-tissue variations in the different compounds using LC-MS/MS analyses. Biomarker biocloud platform (www.biocloud.net) was used to carry out instrument stability after combining the samples. After that, UHPLC system (1290, Agilent Technologies) containing of a TripleTOF 5600 (Q-TOF, AB Sciex) and UPLC BEH Amide column (1.7 μm 2.1*100 mm, Waters) was used to carry out LC-MS/MS analyses. More details regarding LC-MS/MS analyses, metabolites processing, and annotation were documented in previous studies (Fallah et al., 2022; Yuan et al., 2022)

### 2.5 Data analysis

We performed volcano plot analysis using ggtern and grid, an extension of the package ggplot2, to explore the depleted and enriched metabolites in the various compartments under the different treatments. Venn diagrams were also plotted to quantify depleted and enriched metabolites in each compartment (http://bioinfogp.cnb.csic.es/tools/venny/index.html). Interactive networks of metabolites and plant-soil systems were constructed to understand the relationship between metabolites under the different treatments and in both compartments following the approach documented in previous study (Toju et al., 2016). The correlations among bacteria, the targeted metabolites, and soil physico-chemical characteristics were explored using a correlation matrix. All potential pairwise Spearman’s ranks were calculated and visualized using Cytoscape (version 3.6.1). The test data were evaluated using ANOVA and displayed by GraphPad Prism (version 9). Tukey’s HSD test (*p* < 0.05) was adopted to compare the difference between the values of the mean. The rest of the figures used in this study were generated using biomarker biocloud platform (www.biocloud.net).

## 3 Results

### 3.1 Ratoon crop traits and soil physico-chemical characteristics response to organic amendments

**Figure 1:**
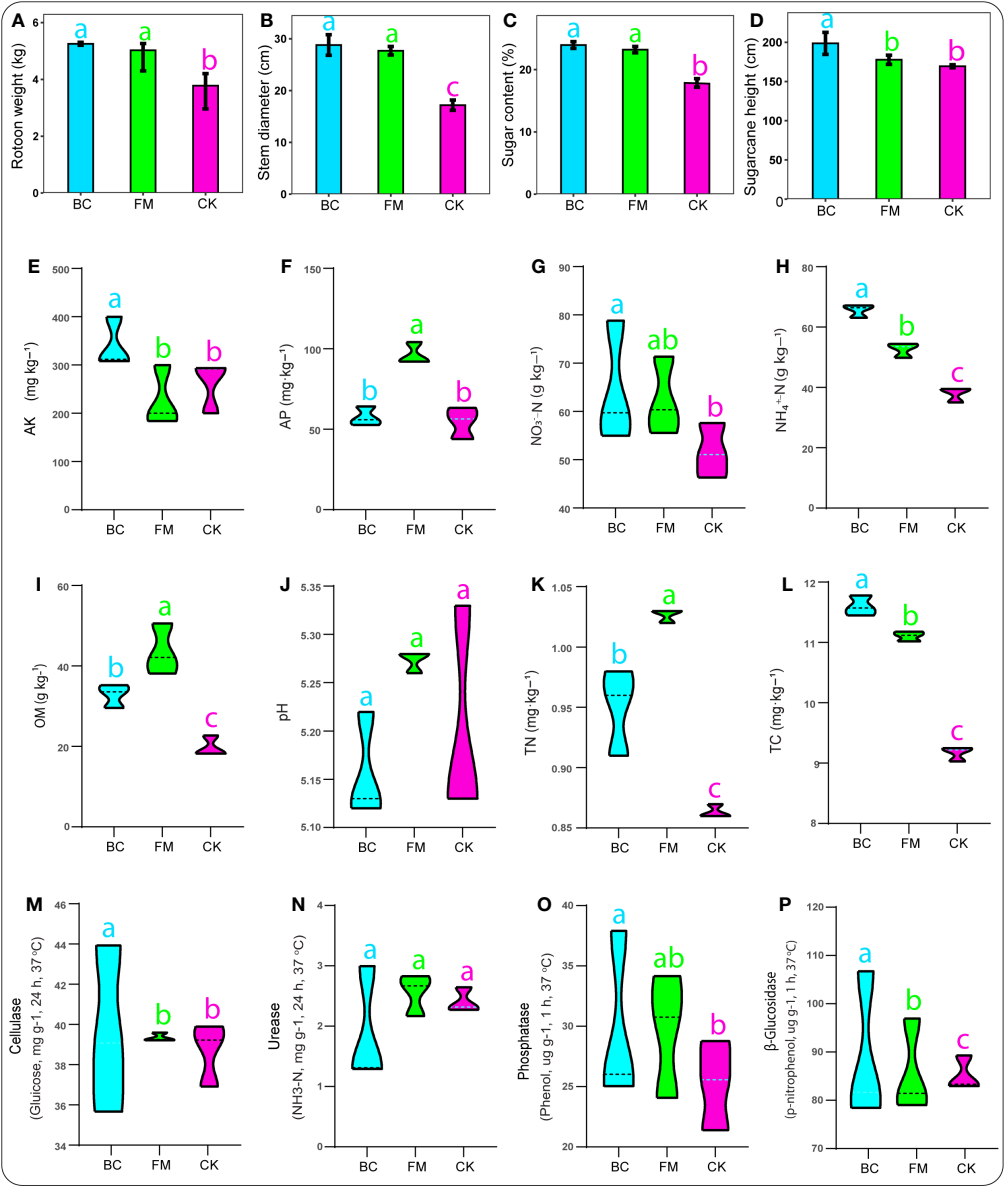
Bar charts revealing crop traits **(A–D)**. Soil physico-chemical characteristics: **(E)** AK, available potassium; **(F)** AP, available phosphorus; **(G)** NO_3_^–^N, nitrate; **(H)** NH_4_^+^-N, ammonium; **(I)** OM, organic matter content; **(J)** pH, potential hydrogen; **(K)** TN, total nitrogen; **(L)**, total carbon, followed by soil enzymes activities **(M–P)**. Graphs with different lowercase letters represent significant differences among treatments (Tukey test, *p* < 0.05).

We observed that the various crop traits were responsive to the different amendments, with BC and FM significantly enhancing (P < 0.05) ratoon weight, stem diameter, and sugar content compared with the CK treatment (**Figures 1A–D**). Additionally, the crop height peaked significantly (P < 0.05) under the BC treatment than the CK treatment (**Figure 1C**).

The effects of both BC and FM on soil physico-chemical characteristics varied distinctly. For instance, soil AK, NO_3_^–^N, cellulase activity, and phosphatase peaked considerably (P < 0.05) under the BC-amended soil compared with the CK treatment (**Figures 1E, G, M, O**). Additionally, soil NH_4_^+^-N, OM, TN, TC, and β-Glucosidase significantly increased (P < 0.05) under both BC and FM-amended soils compared with the CK treatment (**Figures 1H, I, K, L, P**). Soil AP significantly peaked (p < 0.05) under the FM treatment compared with both BC and CK treatments (**Figure 1F**). The BC-amended soil significantly moderated the soil pH compared with the CK treatment (**Figure 1J**). However, urease activity showed no significant difference among the treatments (**Figure 1N**).

### 3.2 Metabolites abundance and composition in the rhizosphere soil and root tissue under the different amendments

**Figure 2:**
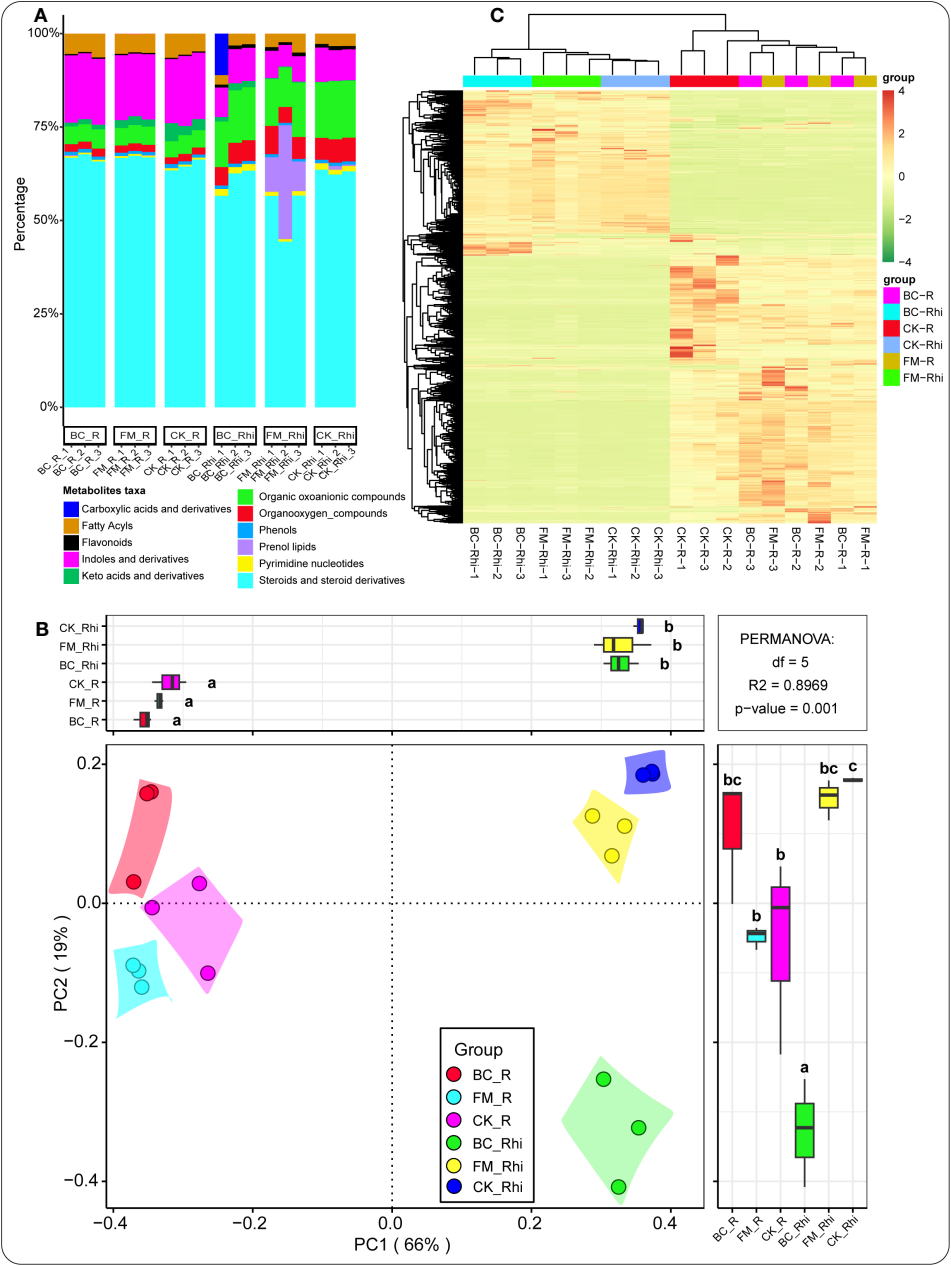
Metabolites relative abundance in both compartments under the different treatments **(A)**. Principal component analysis (PCA) of metabolites in all samples **(B)**. Cluster heat map of metabolites under the different treatments in the various compartments **(C)**. CK-R, control root tissue; CK-Rhi, control rhizosphere soil; BC-R, root tissue of the biochar-amended soil; BC-Rhi, rhizosphere soil of the biochar-amended soil; FM-R, root tissue of the filter mud-amended soil; and FM-Rhi, rhizosphere soil of the filter mud-amended soil. Different lowercase letters denote the different categories or groups of samples.

We identified 11 dominant metabolites taxa with known pathways in the entire taxa, including steroids and steroid derivatives (59.42%), organic oxoanionic compounds (12.25%), indoles and derivatives (9.39%), prenol lipids (5.44%), organooxygen compounds (5.17%), fatty acyls (3.53%), pyrimidine nucleotides (1.21%), carboxylic acids and derivatives (1.05%), keto acids and derivatives (0.84%), flavonoids (0.80%), and phenols (0.80%) (**Figure 2A**, **Table S1**). Principal coordinates analysis (PCoA) was performed to explore the dissimilarity and similarity of metabolite composition in the root tissue and rhizosphere soil under the different treatments. A unique pattern was observed between the root tissue and rhizosphere soil. In the rhizosphere soil, metabolites composition was densely clustered together under the different treatments. In contrast, those in the root tissue exhibited the opposite (**Figure 2B**). Cluster heatmap was further conducted to broaden our understanding of the expression pattern of metabolites in the root tissue and the rhizosphere soil under the different treatments. The result further confirmed that the metabolite distribution patterns were compartment-specific (**Figure 2C**).

### 3.3 Metabolites differential abundance and correlation in both compartments under the different amendments

We later performed K-means clustering to identify the compartment and treatment in which metabolite expression was more pronounced. Noticeably, plant metabolites were significantly expressed in the rhizosphere soil of the BC-amended soil (**Figure S1A**). Venn diagram analysis was adopted to quantify the total number of metabolites unique or common in the various compartments. The analysis revealed that 67 and 47 metabolites were unique to the root tissue of the BC and FM-amended soils compared with the root tissue of the CK treatment, respectively. Additionally, 37 metabolites were unique to the rhizosphere soil of the FM-amended soil compared with the rhizosphere soil of the CK treatment. We also observed that 113 were unique to the rhizosphere soil of the FM-amended soil compared with the rhizosphere soil of the BC-amended soil (**Figure S1B**).

Spearman correlation showed that plant metabolites were specific to the compartments and treatments. Furthermore, metabolites detected in the rhizosphere soils of the different treatments were more prevalent compared with the root tissue (**Figure S1C**). Quality control (QC) of the samples was further performed to validate the result illustrated in **Figure S1C**. The result revealed that the QC samples’ correlation was above 0.9, suggesting that the data generated were valid (**Figure S2**). Pearson correlation coefficient analysis was also conducted to detect the metabolites that were functionally coregulated or related to each other in both compartments under the different treatments. Metabolites-root associations in the same compartments under the different treatments showed unique profiles (**Figure S3A-D**). **Figure S3A** demonstrated that a significant number of metabolites in the rhizosphere soils of both CK treatment and BC-amended soils were significantly and positively correlated (P < 0.05), accounting for 136 positive and 74 negative correlations. On the contrary, a significant number of metabolites in the rhizosphere of the CK treatment and the FM-amended soil showed significant negative correlations, accounting for 111 positive and 98 negative correlations (**Figure S3B**). Likewise, many metabolites in the root tissue of the CK treatment and the BC-amended soils displayed similar patterns (**Figure S3C**), accounting for 110 positive and 100 negative correlations. A significant number of metabolites in the root tissue of the CK treatment and the FM-amended soils exhibited significant positive correlations (P < 0.05), accounting for 146 positive and 64 negative correlations (**Figure S3D**).

### 3.4 Enriched and depleted metabolites in both compartments under the different amendments

**Figure 3:**
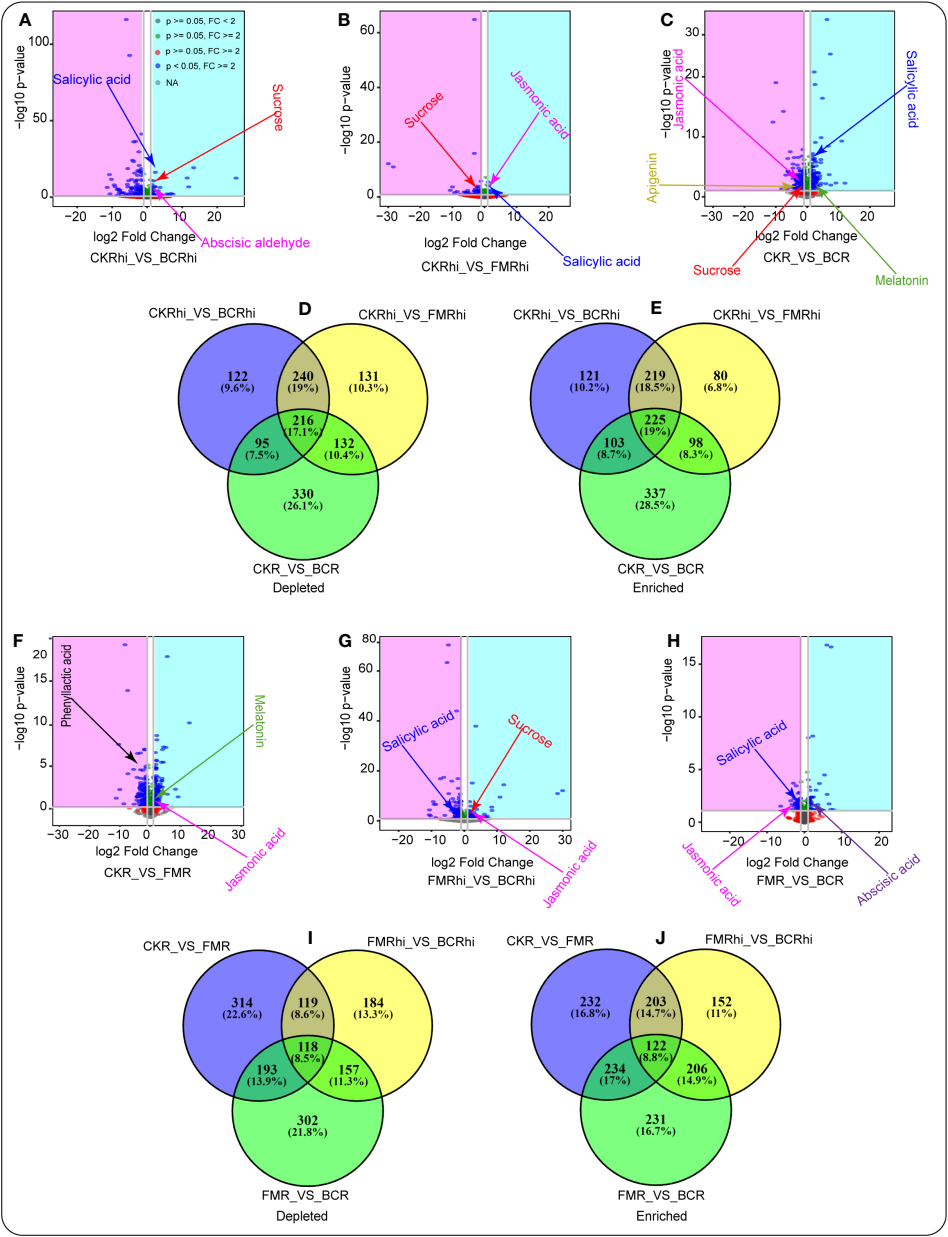
Volcano plots illustrating the depleted (pink) (blue) and enriched metabolites in the same plant compartment under the different amendments **(A–C, F–H)**. Venn diagrams revealing the depleted metabolites **(D, I)** and enriched metabolites **(E, J)** that are overlapping and unique in one compartment relative to the other. Each point in the volcano chart represents a metabolite.

Volcano plot analysis was performed to detect the enriched or depleted metabolites in the same compartment under the different treatments (3A-C, F-H). We observed that several essential secondary metabolites performed better in the amended soils relative to the CK treatment. Salicylic acid (SA), abscisic aldehyde, and sucrose enriched in the rhizosphere soil of the BC amended soil compared with the rhizosphere soil of the CK treatment (**Figure 3A**, **Table S2**). JA and SA revealed a similar pattern in the rhizosphere soil of FM-amended soil compared with the rhizosphere soil of the CK treatment. At the same time, sucrose was depleted in the rhizosphere soil of the CK treatment compared with the rhizosphere soil of the FM-amended soil (**Figure 3B**, **Table S3**). Moreover, melatonin and SA exhibited a similar trend in the root tissue of the BC-amended soil relative to those identified in the root tissue of the CK treatment. Apigenin and JA were depleted in the root tissue of the CK treatment relative to those detected in the root tissue of the BC-amended soil (**Figure 3C**, **Table S4**).

Venn diagram was later adopted to quantify the total depleted (3D and I) and enriched (3E and J) metabolites in each compartment relative to those in another compartment. We noticed that 122 (9.6%) depleted metabolites were unique in the rhizosphere soil of the CK treatment relative to those identified in the rhizosphere soil of the BC amendment. Moreover, 131 (10.3%) depleted metabolites were unique in the CK rhizosphere soil compared with the rhizosphere soil of the FM amendment. Likewise, 330 (26.1%) depleted metabolites were unique in the root tissue of the CK treatment relative to those detected in the root tissue of the BC amendment (**Figure 3D**, **Tables S2**-**4**). Venn diagram also revealed that 121 (10.2%) enriched metabolites were unique in the rhizosphere soil of the BC amended soil compared with the rhizosphere soil of the CK treatment. It was also noticed that 80 (6.8%) enriched metabolites were unique in the rhizosphere soil of the FM-amended soil compared with the rhizosphere soil of the CK treatment. Furthermore, 337 (28.5%) enriched metabolites were unique in the root tissue of the BC amendment relative to those identified in the root tissue of the CK treatment (**Figure 3E**, **Tables S2**-**4**).

Similarly, volcano plot analysis showed that melatonin and JA were enriched in the root tissue of the BC amendment compared with those detected in the root tissue of the CK treatment. PLA was depleted in the root tissue of the CK treatment compared with the root tissue of the FM amendment (**Figure 3F**, **Table S5**). In addition, JA and sucrose were enriched in the rhizosphere soil of the BC-amended soil compared with the rhizosphere soil of the FM amendment. SA was depleted in the rhizosphere soil of the FM amendment compared with the rhizosphere soil of the BC amendment (**Figure 3G**, **Table S6**). Abscisic acid was enriched in the root tissue of the BC-amended soil compared with the root tissue of the FM amendment. SA and JA were depleted in the root tissue of the FM amendment relative to those identified in the root tissue of the BC-amended soil (**Figure 3H**, **Table S7**).

Venn diagram also demonstrated that 314 (22.66%) depleted metabolites were unique in the root tissue of the CK treatment compared with the root tissue of the BC amended soil. Moreover, 184 (13.3%) depleted metabolites were unique in the root tissue of the CK treatment compared with the root tissue of the FM-amended soil. Similarly, 302 (21.8%) depleted metabolites were unique in the root tissue of FM-amended soil compared with the root tissue of the BC-amended soil (**Figure 3I**, **Tables S5**-**7**). Venn diagram also revealed that 232 (16.8%) enriched metabolites were unique in the root tissue of the FM-amended soil compared with the root tissue of the CK treatment. We also observed that 152 (11%) enriched metabolites were unique in the rhizosphere soil of the BC amended soil compared with the rhizosphere soil of the FM treatment. Furthermore, 231 (28.5%) enriched metabolites were unique in the root tissue of the BC-amended soil relative to those detected in the root tissue of the FM-amended soil (**Figure 3J**, **Tables S5**-**7**).

### 3.5 KEGG pathways of annotated and enriched metabolites in both compartments under the different amendments

**Figure 4:**
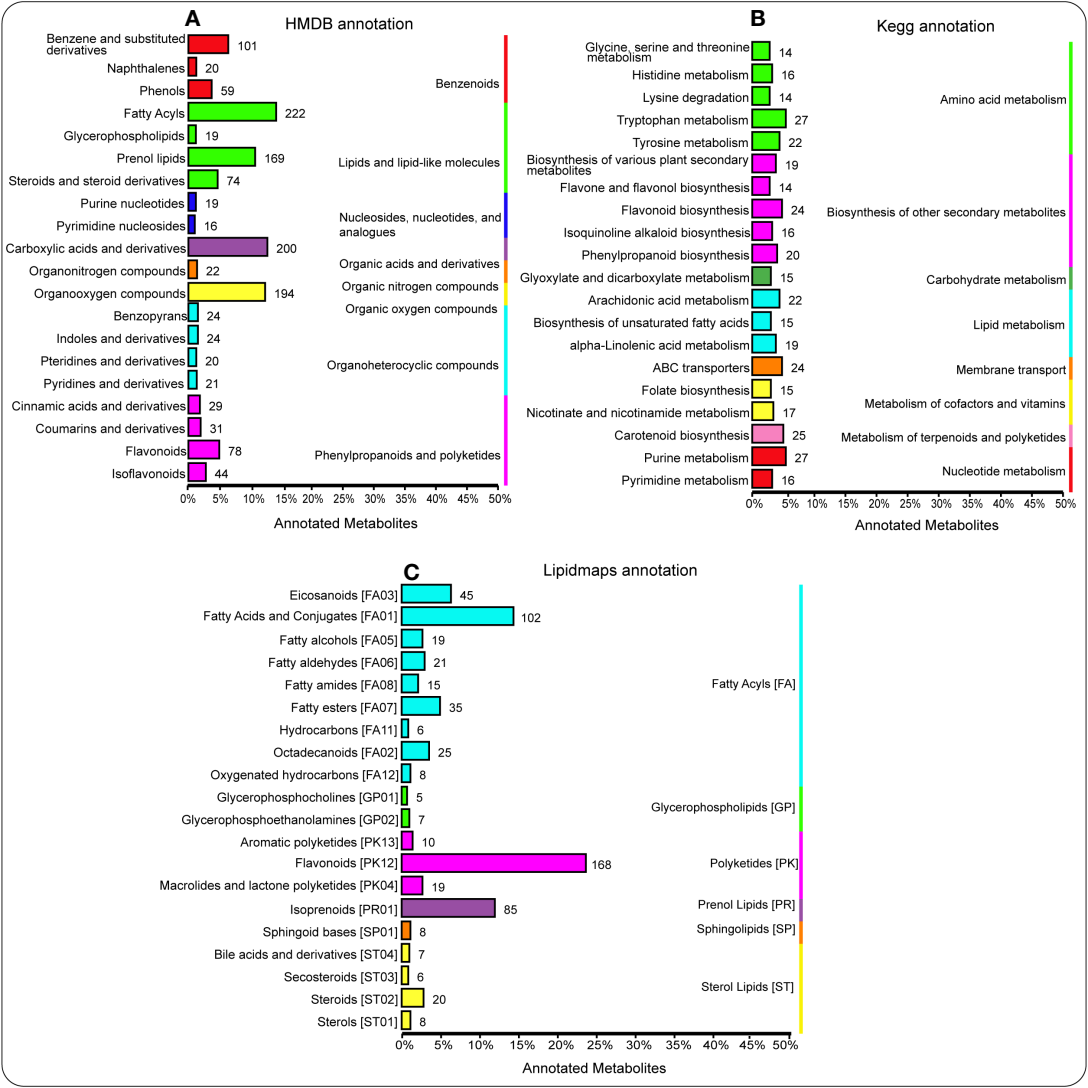
HMDB database annotations illuminate the hierarchy classification information corresponding to the superclass and class information of the HMDB database. The column length represents the number of metabolites annotated to this classification **(A)**. Annotation of metabolites using the KEGG pathway. The entries under the same box in the figure represent the hierarchical classification notes of the KEGG pathway, corresponding to KO pathway level 1 and KO pathway level 2. The column length represents the number of metabolites annotated to this pathway **(B)**. Lipidmaps database annotation showing the lipid maps hierarchy classification information, corresponding to the category and main class information of the lipid maps database. The column length represents the number of metabolites annotated to this classification **(C)**.

Here, Human Metabolome Database (HMDB) revealed that lipids and lipid-like molecules (e.g., fatty acyls, prenol lipids, steroids, and steroid derivatives), organic acids and derivatives (e.g., carboxylic acids and derivatives), and organic oxygen compounds (e.g., organooxygen compounds) were considerably enriched (P < 0.05) (**Figure 4A**). Additionally, **Figure 4B** showed that there were more metabolites enriched in amino acid metabolism (e.g., tryptophan metabolism and tyrosine metabolism), nucleotide metabolism (e.g., purine metabolism), and metabolism of terpenoids and polyketides (e.g., carotenoid biosynthesis). Besides, LIPID MAPS showed that polyketides (e.g., flavonoids) and fatty acyls (e.g., fatty acids and conjugates, eicosanoids, and fatty esters) were significantly enriched (P < 0.05) (**Figure 4C**).

Bar plot of enriched KEGG signaling pathways showed that carotenoid biosynthesis and biosynthesis of unsaturated fatty acids were significantly enriched (P < 0.05) in the rhizosphere soil of the BC-amended soil and compared with the CK treatment (**Figure S4A**). Moreover, terpenoid backbone biosynthesis, alanine, aspartate and glutamate metabolism, glyoxylate and dicarboxylate metabolism, and phenylpropanoid biosynthesis enriched considerably (P < 0.05) in the rhizosphere soil of the FM-amended soil compared with the rhizosphere soil of the CK treatment (**Figure S4B**).

In the root tissue of the BC-amended soil, histidine metabolism, betalain biosynthesis, etc., were significantly enriched (P < 0.05) compared with the root tissue of the CK treatment (**Figure S4C**). Similarly, flavonoid biosynthesis, flavone and flavonol biosynthesis, glucosinolate biosynthesis, followed by tryptophan metabolism, demonstrated the same pattern in the root tissue of the FM-amended soil compared with the root tissue of the CK treatment (**Figure S4D**).

### 3.6 Soil–plant metabolites interactions in both compartments under the different amendments

**Figure 5:**
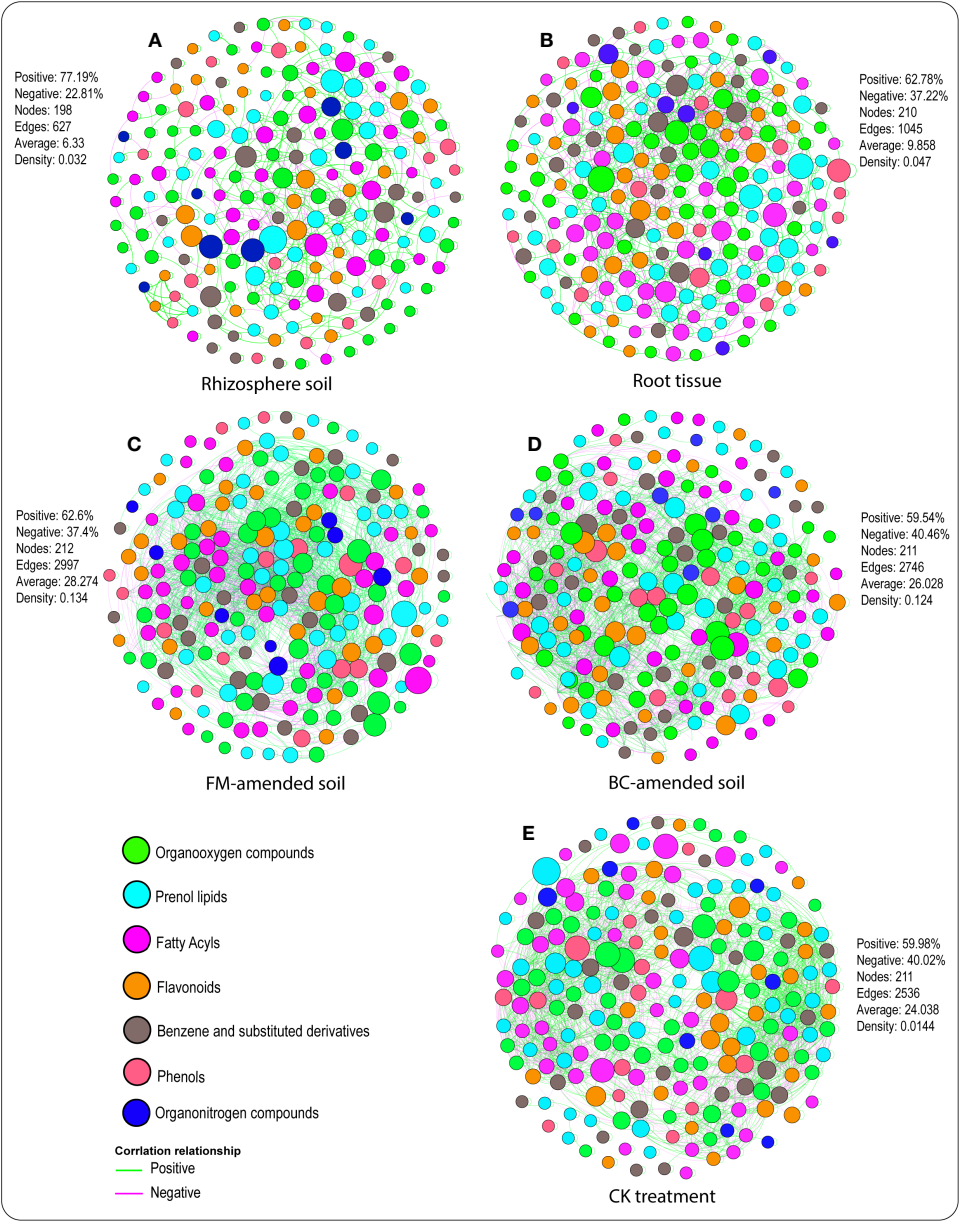
Topological features of networks visualizing soil–plant metabolites association in the rhizosphere soil **(A)**, root tissue **(B)** of the FM-amended soil **(C)**, BC-amended soil **(D)**, and CK treatment **(E)**. A node indicates a root exudate, and green and purple lines denote positive and negative correlations.

Network analysis revealed the interactions between metabolites and plant-soil systems in both compartments (**Figures 5A, B**) under the different treatments (**Figures 5C–E**). Dissimilarities were observed among the topological parameters, namely, the negative and positive interactions, the number of network nodes and edges, etc. For instance, metabolite associations with plant-soil systems in the rhizosphere soil contained a 77.19% positive correlation, 22.81% negative correlation, 198 nodes, 627 edges, 6.33 average connectivity, and 0.032 density (**Figure 5A**). In the root tissue, metabolite associations with plant-soil systems comprised 62.78% positive correlation, 37.22% negative correlation, 210 nodes, 1045 edges, 9.858 average connectivity, and 0.047 density (**Figure 5B**). Furthermore, 62.6% positive correlation, 37.4% negative correlation, 212 nodes, 2997 edges, 28.274 average connectivity, and 0.134 density were identified in the FM-amended soil (**Figure 5C**). In the BC-amended soil, 59.54% positive correlation, 40.46% negative correlation, 211 nodes, 2746 edges, 26.028 average connectivity, and 0.124 density were detected (**Figure 5D**). Whereas the CK treatment accounted for 59.98% positive correlation, 40.02% negative correlation, 211 nodes, 2536 edges, 24.038 average connectivity, and 0.014 density (**Figure 5E**).

### 3.7 Bacteria abundance and community composition response to long-term organic amendments

Meanwhile, we also identified and explored the abundance of bacteria. The entire sample was mainly dominated by a number of bacteria phyla, including Proteobacteria, Myxococcota, Bacteroidota, Acidobacteriota, followed by Actinobacteriota, Gemmatimonadota, Patescibacteria, Firmicutes, Nitrospirota, and Bdellovibrionota (**Figure S5A**). These bacteria community compositions exhibited a tissue-specific pattern under the different amendments (**Figure S5B**).

We later conducted BugBase functional analysis to quantify the effect of the BC and FM amendments on the phenotypes of potentially pathogenic (**Figures S5C, D**). The analysis showed that the relative abundance of potentially pathogenic bacteria in the root tissue and rhizosphere soil of CK treatment was more pronounced compared with the BC and FM-amended soils (**Figure S5C**). We also performed BugBase functional analysis to confirm further the pattern observed using the OTUs distributions of potentially pathogenic (**Figure S5D**). We observed that the OTUs distributions of pathogenic bacteria were significantly higher in the CK treatment compartments compared with the compartments of the BC and FM-amended soils. This behavior was primarily driven by bacteria belonging to f:Sinobacteraceae and f:Xanthomonadaceae in both compartments, followed by Devosia and Burkholderia in the root tissue.

### 3.8 Metabolites associations with beneficial bacteria and soil physico-chemical characteristics

**Figure 6:**
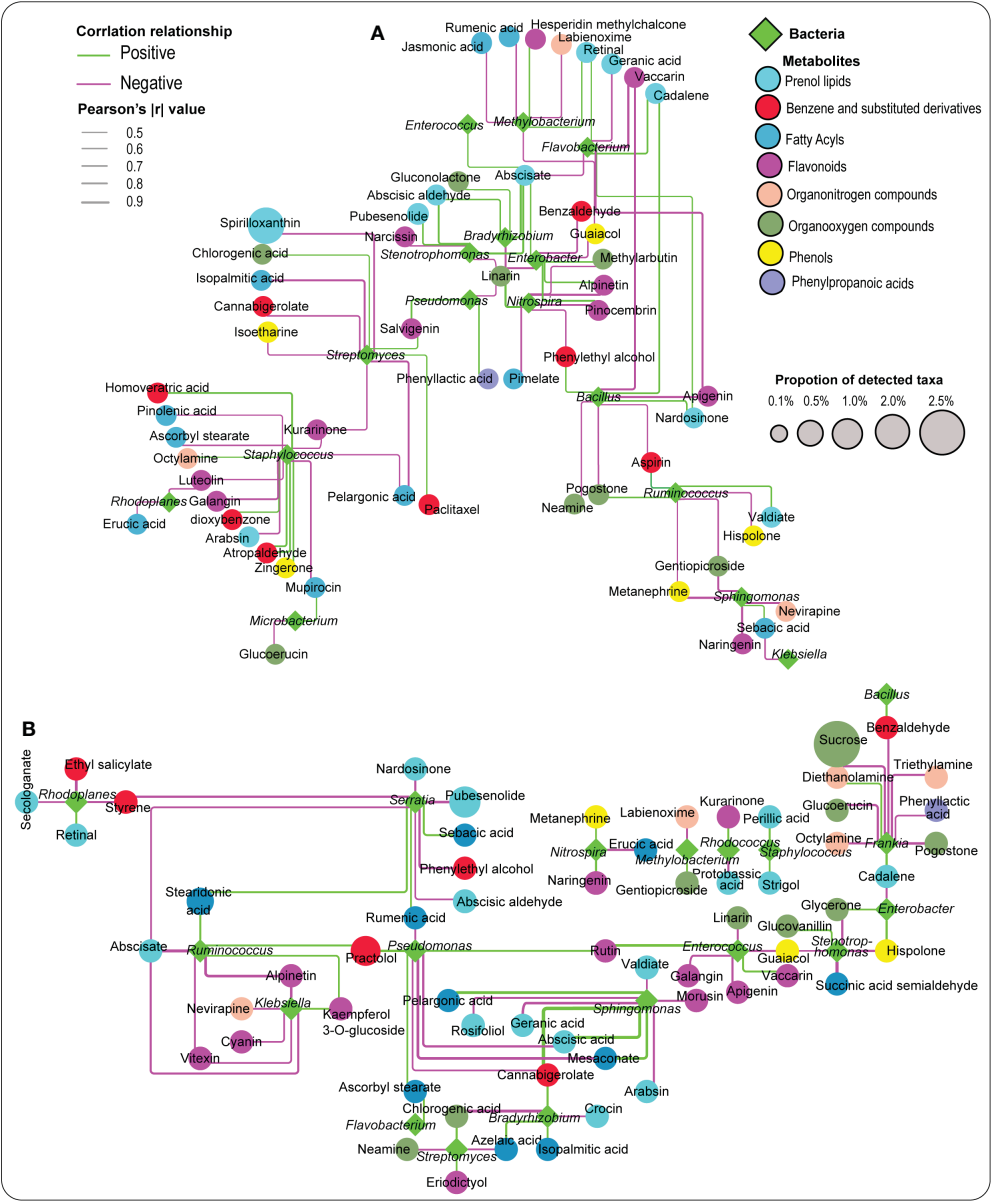
Network analysis illustrating the relationship between metabolites and selected bacteria detected in the rhizosphere soil **(A)** and the root tissue **(B)**. Pink and green lines represent negative and positive correlations, respectively.

Bacteria genera were later used to explore their associations with essential metabolites, including abscisic aldehyde, PLA, ABA, apigenin, JA, etc. (**Figures 6A, B**, **Tables S8** and **S9**). Remarkably, a number of these essential metabolites demonstrated distinct patterns with these bacteria. For example, PLA exhibited a strong positive association with *Pseudomonas*, while abscisic aldehyde demonstrated a significant positive association with *Bradyrhizobium* and *Stenotrophomonas* in the rhizosphere soil (**Figure 6A**, **Table S8**). In the root tissue, ABA, naringenin, rumenic acid, benzaldehyde, and azelaic acid responded strongly and positively to *Sphingomonas*, *Nitrospira*, *Serratia*, *Bacillus*, and *Bradyrhizobium*, respectively. At the same time, rutin revealed a significant positive relationship with *Pseudomonas* and *Enterococcus* (**Figure 6B**, **Table S9**).

We also tested the interactions between soil physico-chemical characteristics and beneficial bacteria (**Tables 1**, **2**). A number of essential bacteria exhibited significant positive associations with soil physico-chemical characteristics in both compartments. *Bradyrhizobium*, *Streptomyces*, and *Rhodococcus* revealed a strong positive correlation with soil AP in the root tissue. Moreover, *Enterococcus* showed the same pattern with cellulase activity, while *Rhodoplanes* demonstrated a similar trend with phosphates and urease activity (**Table 2**), suggesting that these bacteria played a crucial role in promoting vital soil nutrients. *Streptomyces*, *Staphylococcus*, and *Nitrospira* in the rhizosphere soil were significantly and positively correlated with essential soil nutrients, including soil AK, TN, and AP, respectively. Likewise, *Streptomyces* responded strongly and positively to soil NH_4_^+^-N, while *Bradyrhizobium* showed a strong positive relationship with soil AP and OM. Moreover, *Stenotrophomonas* and *Staphylococcus* displayed a similar pattern with vital soil nutrients, namely, NH_4_^+^-N and NO_3_^–^N, respectively. Besides, *Staphylococcus* and *Streptomyces* had a significant positive relationship with soil NO_3_^–^N and β-Glucosidase, respectively (**Table 3**).

**Table 2:** Beneficial bacteria detected in the root tissue correlate with soil physico-chemical characteristics.

raw.r	raw.p	Targeted bacteria	Soil properties	Compartment
-0.84	0	*Klebsiella*	OM	Root tissue
0.67	0.05	*Bradyrhizobium*	AP	Root tissue
0.72	0.03	*Streptomyces*	AP	Root tissue
-0.71	0.03	*Rhodococcus*	AP	Root tissue
-0.69	0.04	*Nitrospira*	TC	Root tissue
0.71	0.03	*Enterococcus*	Cellulase activity	Root tissue
-0.71	0.03	*Rhodococcus*	Cellulase activity	Root tissue
0.68	0.04	*Rhodoplanes*	Phosphates	Root tissue
-0.72	0.03	*Enterobacter*	Urease activity	Root tissue
0.78	0.01	*Rhodoplanes*	Urease activity	Root tissue

**Table 3:** Beneficial bacteria detected in the rhizosphere soil correlate with soil physico-chemical characteristics.

raw.r	raw.p	Targeted bacteria	Soil properties	Compartment
0.85	0	*Streptomyces*	AK	Rhizosphere soil
-0.81	0.01	*Staphylococcus*	OM	Rhizosphere soil
0.78	0.01	*Staphylococcus*	TN	Rhizosphere soil
-0.78	0.01	*Staphylococcus*	TC	Rhizosphere soil
-0.8	0.01	*Rhodoplanes*	NO_3_^–^N	Rhizosphere soil
0.75	0.02	*Nitrospira*	AP	Rhizosphere soil
-0.72	0.03	*Nitrospira*	OM	Rhizosphere soil
0.72	0.03	*Streptomyces*	NH_4_^+^-N	Rhizosphere soil
0.68	0.04	*Bradyrhizobium*	AP	Rhizosphere soil
0.69	0.04	*Bradyrhizobium*	OM	Rhizosphere soil
-0.68	0.04	*Streptomyces*	TC	Rhizosphere soil
0.69	0.04	*Stenotrophomonas*	NH_4_^+^-N	Rhizosphere soil
-0.67	0.05	*Bradyrhizobium*	NH_4_^+^-N	Rhizosphere soil
0.67	0.05	*Staphylococcus*	NO_3_^–^N	Rhizosphere soil
0.69	0.04	*Streptomyces*	β-Glucosidase	Rhizosphere soil
-0.69	0.04	*Ruminococcus*	β-Glucosidase	Rhizosphere soil

Correspondingly, the associations between soil physico-chemical characteristics and the selected metabolites were investigated (**Tables S10**, **S11**). Interestingly, a number of plant essential metabolites, including apigenin, were significantly and positively correlated with crucial soil nutrients, namely, TN and NO_3_^–^N. PLA and naringenin exhibited a similar pattern with soil OM and urease activity in the root tissue (**Table S4**).

In the rhizosphere soil, abscisic aldehyde revealed a strong positive correlation with soil NH_4_^+^-N, urease activity, and AP, while sucrose responded strongly and positively to soil pH and phosphates activity (**Table S5**). This behavior suggests that plant metabolites played a crucial role in enhancing vital soil nutrients.

## 4 Discussion

Studies have shed light on the decline in ratoon crop growth, development, and productivity, mainly due to nutrient depletion and pest and disease outbreaks (Xu et al., 2021; Fallah et al., 2023). We recently reported that BC was key in rejuvenating ratoon crop traits (Fallah et al., 2023). Here, ratoon crop traits under the sustained effect of BC and FM-supplemented soils peaked significantly, largely due to the regulatory effects of these amendments on pathogenic bacteria and stress-regulating metabolites, which draws parallels with previous findings (Gomathi, 2013).

Soil physico-chemical characteristics, including soil AK, NO_3_^–^N, cellulase activity, and phosphatase, peaked under the BC-amended soil compared with the CK treatment. Whereas soil NH_4_^+^-N, OM, TN, TC, and β-Glucosidase marked a significant increase under the BC and FM-amended soils compared with the CK treatment. It is worth mentioning the BC-amended soil was worthy of moderating soil pH compared with the CK treatment, suggesting that organic amendment can exhibit a competitive advantage by boosting soil fertility and regulating soil stresses (Dawan and Ahn, 2022; Pang et al., 2022). The mechanism underpinning this behavior could be ascribed to the ability of organic amendments such as BC and FM to promote essential metabolites (Li et al., 2020), including melatonin, JA, PLA, abscisic aldehyde, and ABA, which, in turn, stimulate vital soil bacteria (Hassan et al., 2017), thereby boosting soil health and fertility. This finding was further validated by the results observed in **Tables 1** and **2**, including **Tables S4**, **S5**. Our finding draws parallel with the study conducted by Li et al. (2020), wherein they documented that the high presence of starch and sucrose metabolism under a BC-amended soil promoted microbial functions, thereby enhancing soil health and enhancing soil nutrients (e.g., dissolved organic carbon and enzyme activities).

Organic soil amendments such as BC and FM can considerably affect metabolite abundance and composition (Wang et al., 2022), precipitating beneficial soil bacteria and eventually promoting soil health, fitness, and fertility (Canarini et al., 2019). Rusli et al. (2022) conducted a six-month greenhouse study to evaluate the performance of metabolites in *Melastoma malabathricum* L. plants subjected to BC-amendment. They reported that the total contents of anthocyanin, phenolic, and flavonoid in the different compartments of *Melastoma malabathricum* L. were more pronounced under a BC-amended soil. Here, **Figures 3A–C, F** revealed that long-term BC and FM amendments enriched a number of stress-regulating compounds in the root tissue (e.g., melatonin, SA, and JA) and rhizosphere zone (e.g., abscisic aldehyde, sucrose, JA, and SA) of the BC and FM-amended soils, respectively. In agricultural soil, melatonin acts as an antioxidant and is crucial in alleviating toxic oxygen and nitrogen species. Melatonin usage is deemed an alternative and inexpensive strategy to enhance crop tolerance against abiotic stressors, namely, pH, heavy metals, and salinity (Moustafa-Farag et al., 2020), evident by the trend observed in **Figure 1F**. Likewise, JA has received extensive research attention as a potential regulator of biotic stresses (pathogen, herbivore, and insect) and abiotic stresses (heavy metal toxicity, drought, salt, heat, and cold) (Wang et al., 2021). We assumed these crucial stress-regulating metabolites play distinct roles in regulating soil biotic and abiotic stresses, evident by results shown in **Figure 1F**, including **Figure S5C, D**. Moreover, BC distinct properties stimulated plant roots to excrete more metabolites, which, in turn, stimulated essential soil bacteria (e.g., *Pseudomonas*, *Bacillus, Sphingomonas*, and *Bradyrhizobium*) (Wang et al., 2022), thereby enhancing the overall condition of the soil, as shown in **Tables 1** and **2**, as well as **Tables S4**, **S5**.

Plant metabolites can trigger the proliferation of a specific group of microbes by serving as an energy source and/or carbon to expedite the metabolism of microbes. In return, the associated microbes provide a plethora of functional capabilities necessary for plant growth and development (Afridi et al., 2022). Essential microbes, such as *Pseudomonas, Sphingomonas*, *Stenotrophomonas, Bradyrhizobium*, etc., may induce a number of regulatory compounds that can ameliorate soil growth conditions by regulating a series of environmental stressors, including drought, phytopathogens suppression, etc. (Köhl et al., 2019). It is worth noting that PLA had a significant positive interaction with *Pseudomonas*, which is widely regarded for its stress-tolerant, biosurfactant, and biofilm potentials (Craig et al., 2021). PLA has the potential to exhibit antimicrobial properties, evident by the finding documented by Paola et al. (2003). These authors proved that PLA triggered an unpredictable delay in the growth of a number of microbial strains, including some mycotoxigenic strains of *Penicillium citrinum*, *Penicillium verrucosum*, and a strain of *Penicillium roqueforti*. The oxidation of abscisic aldehyde is the immediate precursor of ABA biosynthesis and is catalyzed by aldehyde oxidase (Cowan, 2000). The role of ABA in mitigating soil environmental stressors, including pathogens, temperature, heavy metal stress, high salinity, etc., has been well established (Vishwakarma et al., 2017). Abscisic aldehyde in the rhizosphere zone was significantly and positively associated with essential soil bacteria (e.g., *Stenotrophomonas* and *Bradyrhizobium*), while ABA exhibited the same trend with *Sphingomonas* in the root compartment. This phenomenon suggests that the interaction of these stress-regulating compounds with these bacteria played a crucial role in alleviating a series of soil stressors, as these bacteria have demonstrated great potential in remediating environmental stresses and boosting soil fertility and health, as observed in **Figure 1F**. This phenomenon was reinforced by the pattern shown in **Tables 1**, **2**, including **Tables S4** and **S5**, which agrees with the report documented by Hu et al. (2018), in which metabolites regulatory effects on rhizosphere microbes suppressed biotic and abiotic factors and stimulated plant-soil feedback.

## 5 Conclusion

This study demonstrates that long-term BC and FM utilization is one of the surest agriculture strategies to boost ratoon crop traits, soil health, and fertility through the enrichment of stress-regulating metabolites (e.g., ABA, melatonin, JA, abscisic aldehyde, etc.), the suppression of bacterial phenotypes deemed potentially pathogenic.

## Data availability statement

The datasets presented in this study can be found in online repositories. The names of the repository/repositories and accession number(s) can be found in the article/**Supplementary Material**.

## Author contributions

NF: Conceptualization, Data curation, Formal Analysis, Investigation, Methodology, Validation, Visualization, Writing – original draft, Writing – review & editing. ZP: Conceptualization, Data curation, Software, Visualization, Writing – review & editing. ZL: Conceptualization, Methodology, Project administration, Resources, Software, Supervision, Writing – review & editing. WN: Conceptualization, Data curation, Resources, Software, Writing – review & editing. WL: Conceptualization, Data curation, Project administration, Supervision, Writing – review & editing. SM: Conceptualization, Data curation, Investigation, Software, Writing – review & editing. CI: Conceptualization, Resources, Software, Writing – review & editing. HZ: Conceptualization, Funding acquisition, Investigation, Methodology, Resources, Supervision, Validation, Writing – review & editing.
