# Supplementary material for: Sustained organic amendments utilization enhances ratoon crop growth and soil quality by enriching beneficial metabolites and suppressing pathogenic bacteria
Source: Front Plant Sci. 2023 Sep 18;14:1273546. doi: 10.3389/fpls.2023.1273546 (PMC10544933; doi:10.3389/fpls.2023.1273546)
Supplement: Supplementary file 1 [file DataSheet_1.docx]

Supplementary Material

**Metabolites expression trend, differential abundance, and correlation** in both compartments under the different **amendments**


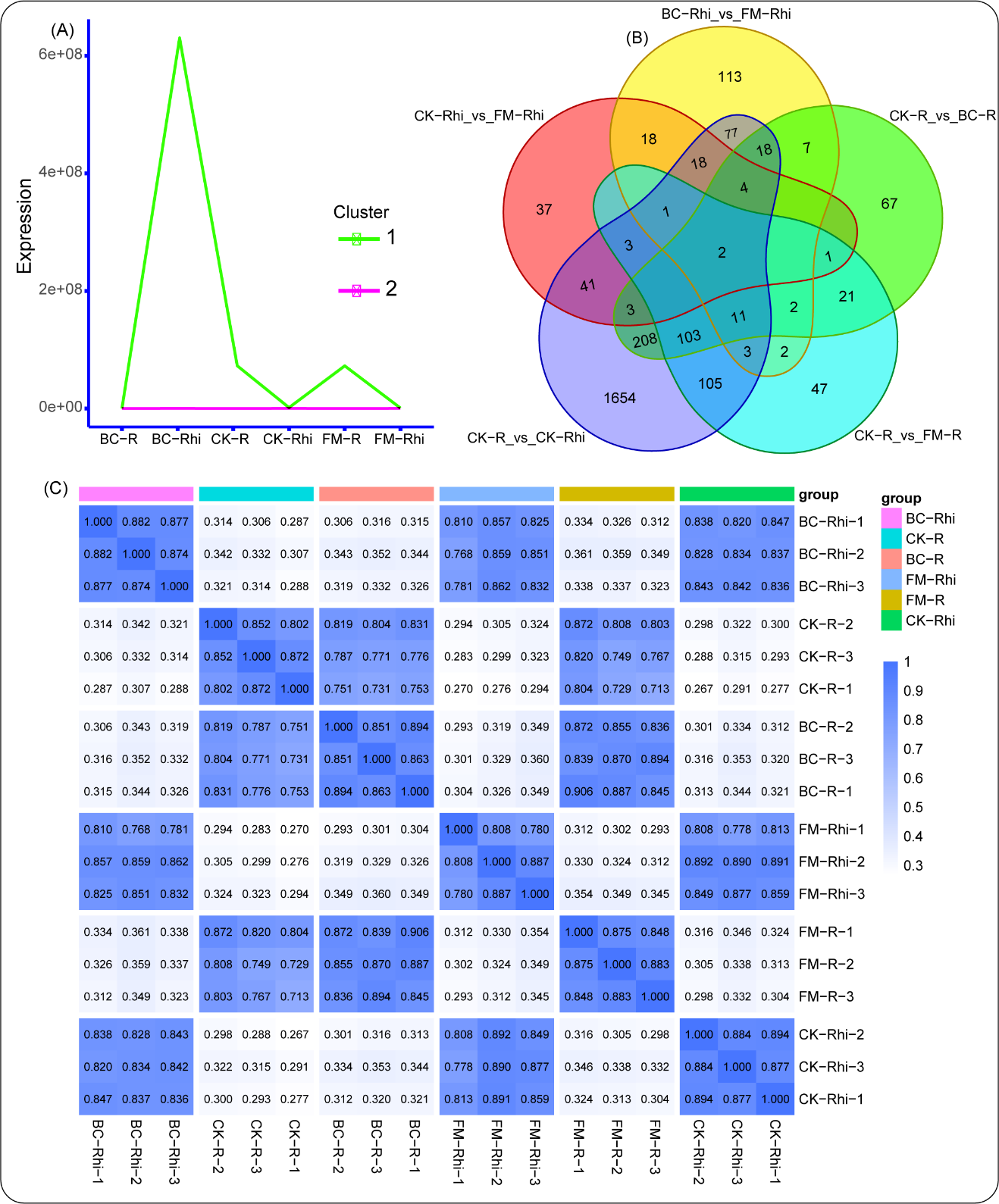


**Figure 1.** K-means clustering trend chart revealing the increase and decrease trends of metabolites under the different treatments in both compartments. The different color lines symbolize the average change trend of root exudate content in each k-means cluster between groups (A). Venn diagram visualization of the unique and overlapped metabolites in both compartments of the different treatments (B). Heat map displaying the Spearman correlation coefficient of pairwise comparison detected between the different samples (C). The longitudinal and horizontal coordinates are sample names, and the color shade characterizes the correlation coefficient r^2^.

**Correlation analysis of QC samples in different treatments**


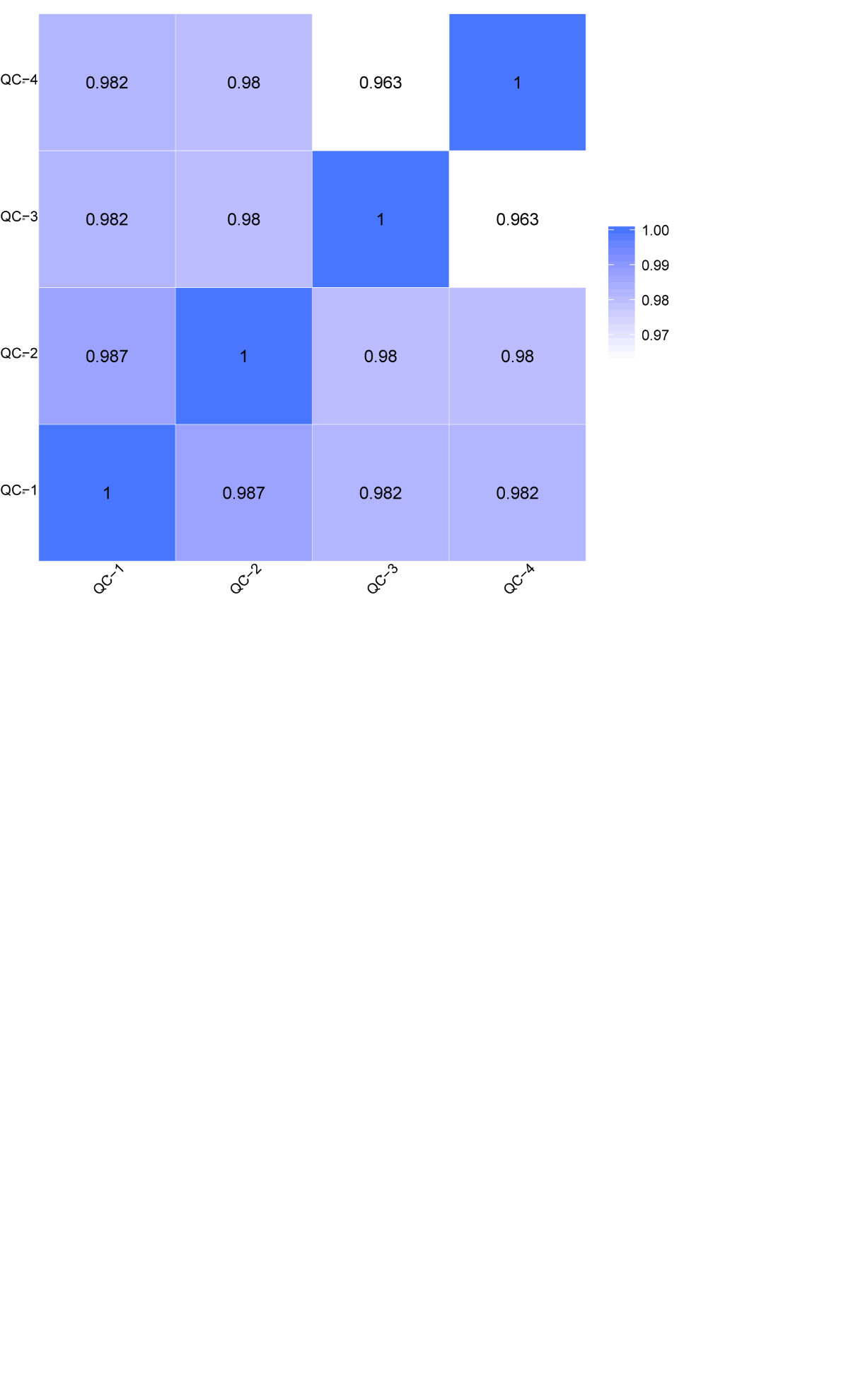


**Figure S2.** Correlation analysis of QC samples. QC samples are a mixture of all tested samples and are used to assess instrument stability. The correlation was above 0.9, signifying that the data generated were valid.

**Metabolites differential abundance correlations in the different compartments under the different treatments**


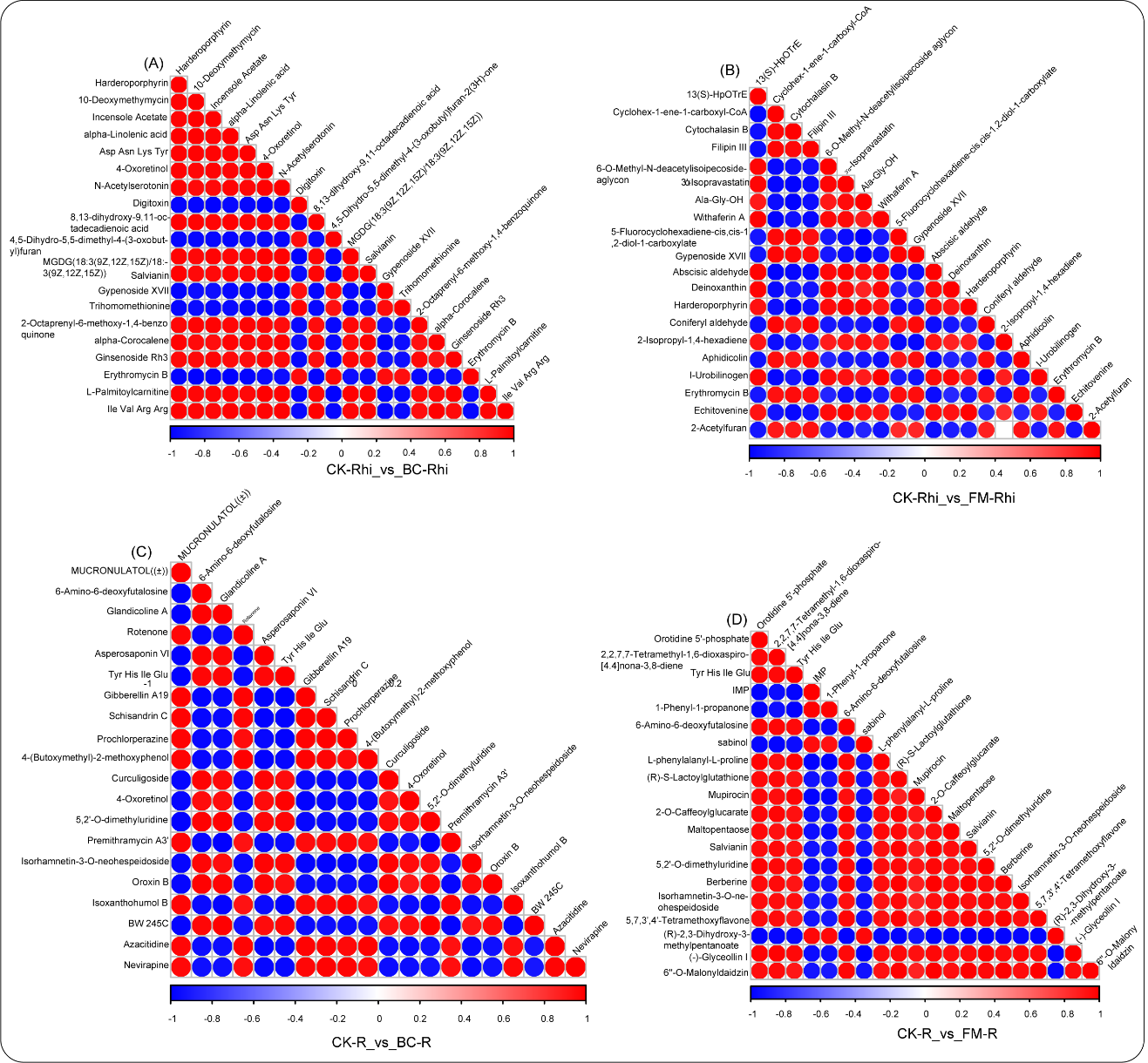


**Figure S3.** Differential metabolic correlation diagram. Note: The highest correlation is 1, which signifies a positive correlation (red); the lowest correlation is -1, which indicates a negative correlation (blue), and the part without color specifies that the significance of the calculated correlation is below the significance threshold. The figures depict the correlation of the top 20 differential root exudates of p-value from small to large by T-test. CK-R, control root tissue; CK-Rhi, control rhizosphere soil; BC-R, root tissue of the biochar-amended soil; BC-Rhi, rhizosphere soil of the biochar-amended soil; FM-R, root tissue of the filter mud-amended soil; and FM-Rhi, rhizosphere soil of the filter mud-amended soil.

**Enriched KEGG signaling pathways of metabolites in the rhizosphere soil**


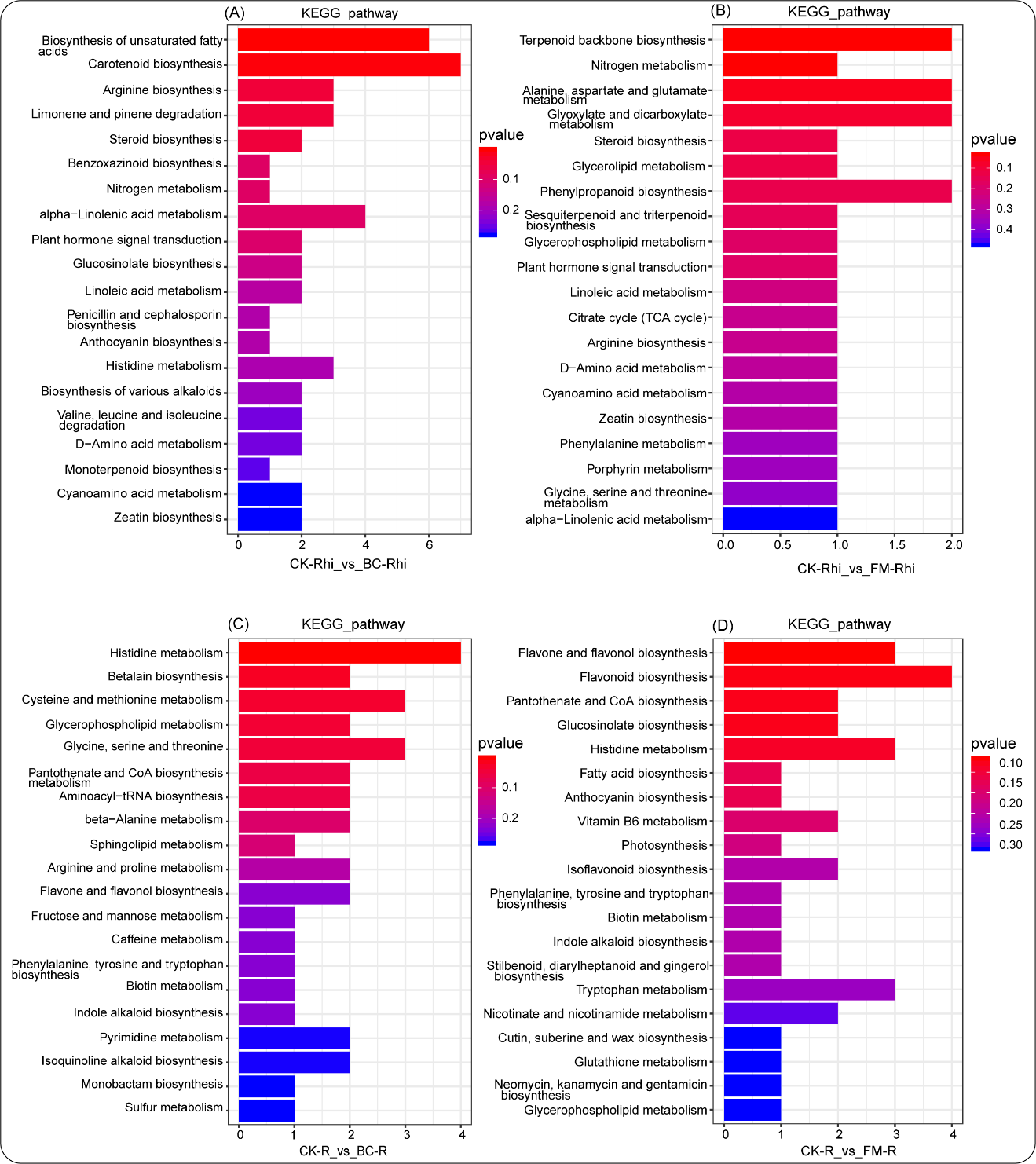


**Figure S4.** Visualization of enriched KEGG signaling pathway of metabolites in the rhizosphere soil (A,B), and the root tissue (C,D) of the different treatments.

B**acteria response to long-term organic amendments in the various soil compartments**


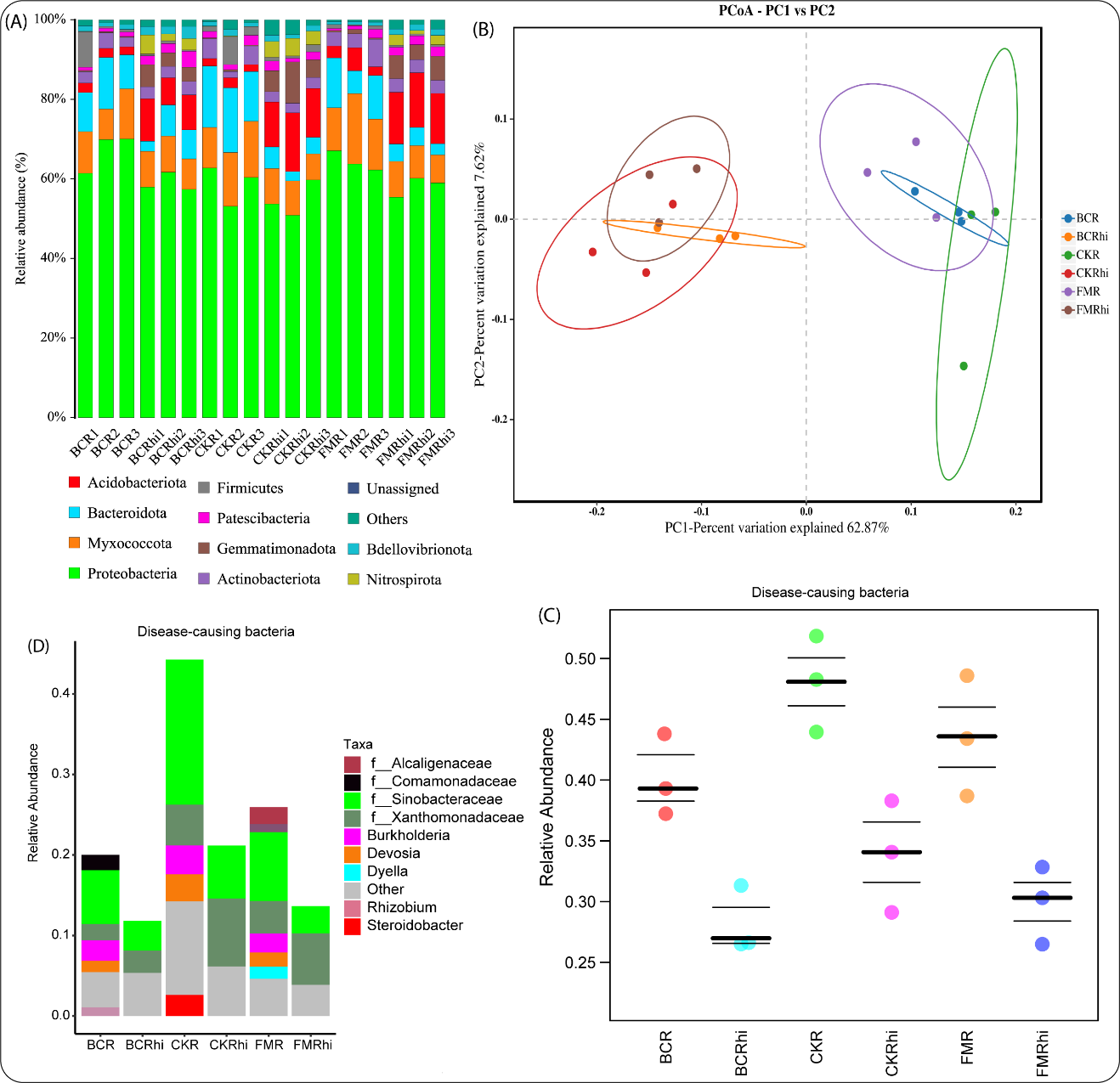


**Figure S5.** Relative abundance of abundant bacteria in the various soil compartments of the different treatments (A). Non-Metric multidimensional scaling (NMDS) analysis based on Bray–Curtis dissimilarities revealing variations in bacterial composition in the different soil compartments (B). BugBase functional analysis illuminating the predicted phenotypes of disease-causing bacteria (C), and its OTUs distribution (D) in the root tissue and rhizosphere soil of the supplemented soil.
